# Influence of Dietary Supplementation for Hyperhomocysteinemia Treatments

**DOI:** 10.3390/nu12071957

**Published:** 2020-06-30

**Authors:** Alessandra Vezzoli, Cinzia Dellanoce, Teresa Maria Caimi, Daniele Vietti, Michela Montorsi, Simona Mrakic-Sposta, Roberto Accinni

**Affiliations:** 1 Institute of Clinical Physiology, National Council of Research (IFC-CNR), ASST Grande Ospedale Metropolitano Niguarda, 20162 Milan, Italy; alessandra.vezzoli@cnr.it (A.V.); dellanoce@ifc.cnr.it (C.D.); roberto.accinni@gmail.com (R.A.); 2 S.S Emostasi, S.C. Ematologia ASST Grande Ospedale Metropolitano Niguarda, 20162 Milan, Italy; teresa.caimi@ospedaleniguarda.it; 3 Driatec srl, Cassina de’ Pecchi, 20060 Milan, Italy; danielevietti@gmail.com; 4 Department of Human Sciences and Promotion of the Quality of Life, San Raffaele Roma Open University, 20122 Milan, Italy; michela.montorsi@uniroma5.it

**Keywords:** magnesium oxoprolinate, folic acid, thiols, homocysteine, randomized study

## Abstract

Hyperhomocysteinemia is recognized as risk factor for cardiovascular and age-associated diseases. Folic acid supplementation efficiently lowers plasma homocysteine (Hcy) levels, but high intake may negatively affect health because of unnatural levels of unmetabolized folic acid in the systemic circulation. Oxoproline (Oxo) provides by glutamic acid production an increase of intracellular folic acid trapping. Aim of this study was to compare the efficacy of three supplementation protocols: (1) traditional therapy (5-methyl-tetrahydrofolate: 15 mg/day); (2) 5 mL/day of Oxo with 300 μg folic acid (oxifolic); (3) 5 mL/day of Oxo alone (magnesio+) in a 90 days randomized trial on thirty-two moderate hyperhomocysteinemic (18.6 ± 2.4 μmol·L^−1^) patients (age 48 ± 14 years). Thiols: cysteine (Cys), cysteinylglycine (Cys–Gly) and glutathione levels were assessed too. Every supplementation induced significant (*p* range <0.05–0.0001) reductions of Hcy level and Cys concentration after the three protocols adopted. Otherwise glutathione concentration significantly increased after oxifolic (*p* < 0.01) and traditional (*p* < 0.05) supplementation. The integration of Oxo resulted an interesting alternative to traditional therapy because absence or minimal number of folates in the integrator eliminates any chance of excess that can constitute a long-term risk.

## 1. Introduction

**Figure 1:**
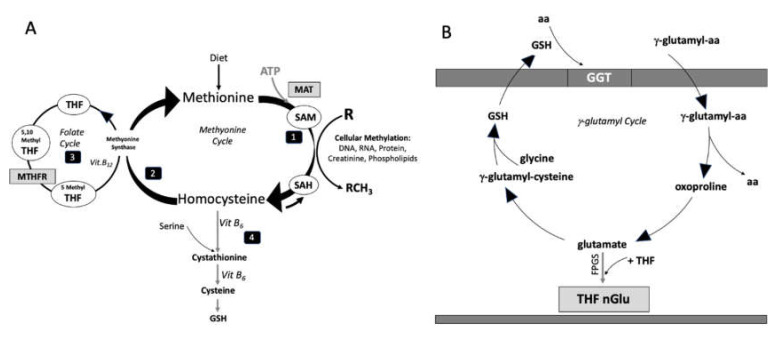
In (**A**): Interactions of methionine cycle, folate cycle, transsulfuration and methylation pathway. (1) Methionine is converted to S-adenosylmethionine (SAM) by methionine adenosyltransferase (MAT). SAM serves as methyl-donor in various cellular methylations; (2) the pathway of remethylation of Hcy to methionine by (3) folate cycle requires the presence of vitamin B12 as coenzyme of the methionine synthase reaction that transfers the one-carbon methyl group from 5-methyl-tetrahydrofolates (THF). MTHFR: methylene-tetrahydrofolate reductase; (4) in the transsulfuration pathway, Hcy reacts with serine to form cystathionine converted in cysteine with the reaction’s vitamin B6 dependent. Cysteine may be in turn be incorporated into glutathione. In (**B**): γ-glutamyl cycle. Glutamyl transpeptidase (GGT) transfers glutamyl aa from blood into cell where oxoproline and glutamate are formed. Thereafter folylpoly-γ-glutamate (FPGS), an ATP-dependent ligase, catalyzes the addition of a polyglutamate tail to reduced folates, one glutamate residue after the other.

Homocysteine (Hcy) is a cysteine homolog, an intermediate product of methionine metabolism and lies at the crossroads of several metabolic pathways. The plasma levels of Hcy are controlled by two metabolic pathways: (1) remethylation of Hcy to methionine, which requires the presence of folic acid and vitamin B12 as coenzymes and occurs within the cell and in all body tissues; (2) transsulfuration of Hcy to cysteine, which requires vitamin B6 (Figure 1A).

Elevations in plasma Hcy concentration (HHcy) can occur due to impairment of enzymes involved in homocysteine and B vitamins metabolism. The decline in renal functions, nutritional deficiencies, lifestyle conditions (physical inactivity, smoking, alcohol consumption) or other factors including use of drugs cause elevation of homocysteine too. Abnormal accumulation of homocysteine is a risk factor of cardiovascular [1], neurodegenerative and chronic kidney disease. Indeed, mildly elevated plasma homocysteine levels are an independent risk factor for atherothrombotic vascular disease in the coronary, cerebrovascular and peripheral arterial circulation inducing endothelial dysfunction [2]. Experimental evidence suggests that the atherogenic propensity associated with HHcy results from endothelial dysfunction and injury followed by platelet activation and thrombus formation [3,4].

Moreover, HHcy would be associated with the shortening of telomeres and hence the cellular aging processes [5]. In this process, folic acid plays a key role, in fact the hypermethylation induced by this vitamin, can reverse this cell senescence. Hcy may accelerate the senescence of endothelial progenitor cells or endothelial cells themselves, by inactivating telomerases and shortening telomeres resulting in circulatory system damage [6,7].

The treatment of HHcy varies with the underlying cause; however, vitamin supplementation (with folic acid, pyridoxine, and vitamin B12) is generally effective in reducing homocysteine concentration.

The Framingham study demonstrated that plasmatic Hcy concentration is inversely correlated to the assumption of folates and group B vitamins [8]. These vitamins are present mainly in green vegetables and meat. However, only a fraction of the folic acid content of food is biologically available, and it is further significantly reduced during cooking and from exposure to light. For these reasons, vitamin supplementation is preferable. The synthetic form of vitamin B9, known as folic acid, has a higher bioavailability being rapidly absorbed across the intestine [9] and better stability to heat, compared to the natural form that is known as folate [10]. Moreover, the folate receptor has a higher affinity for folic acid than for methyl-tetrahydrofolates (5MTHF) the main form of folate that occurs in the blood.

5MTHF transferred to the tissues, penetrating into the cells, is converted back into THF, trapped and cannot recross the cell membranes. THF keeps individual one-carbon (-CH3, -CH2- -CHO, etc.) from certain amino acids and then gives the one-carbon unit to other molecules in reactions catalyzed by different enzymes. Folate, as carriers of one-carbon unit, can be considered essential in the cycles of synthesis and methylation of DNA, proteins and neurotransmitters (see Figure 1A). The sulfur amino acid Hcy is an essential intermediate of these cycles.

Supplementation of folic acid has been demonstrated to be efficient in lowering mildly elevated plasma homocysteine levels, in reversing homocysteine-induced impairment of endothelium-dependent vasoreactivity and in preventing cardiovascular diseases [11,12]. Nevertheless, recent studies on folic acid supplementation showed the toxicities and health consequences related to over-supplementation. It is a fact that high folic acid intake may negatively affect health due to fortification or vitamin supplementation. Folate may influence the development of cancer through its role in one-carbon metabolism and subsequent effects on DNA replication and cell division [13]. Moreover, a high folic acid intake, which is metabolized in liver by dihydrofolate reductase, and the low activity of this enzyme may result in unnatural levels of unmetabolized folic acid entering the systemic circulation [14,15]. The potential risks of this phenomenon appear to be largely ignored [16]. The minimal effective doses of folic acid and pyridoxine have not yet clearly been determined. The conventional treatment of HHcy consists in 5–15 mg daily of folic acid, while 0.4 mg daily are sufficient for RDA [13,17]. An indispensable condition for folic acid to be utilized within the remethylation pathway of Hcy is that its cellular uptake must be irreversible. The capacity of cells to accumulate folates is attributable to the activity of folypoly-γ-glutamate synthetase, an ATP dependent ligase, which catalyzes the attachment of 1–7 glutamate residues to THF, one glutamate residue after the other [18,19].

Oxoproline (Oxo), also known as pidolic acid or pyroglutamic acid, is an amino acid naturally produced by cells as intermediate metabolite that acts as a precursor of glutamic acid (Figure 1B). This function of Oxo provides a strong rational as the synthesis of glutamic acid results in an increase of polyglutamate-folate. This is an indispensable condition for the intracellular trapping of folic acid because the long-chain folylpolyglutamates, with long negatively charged tails, are poorly recognized by the membrane carriers responsible for efflux across the cell membrane [19].

Moreover, oxoproline is an intermediate in γ-glutamyl cycle associated with glutathione (GSH) metabolism (see Figure 1B).

Because homocysteine metabolism is associated with oxidative stress [20], production of GSH, one of the principal antioxidant compounds, results very important.

Indeed, GSH, with Hcy and related thiols cysteine (Cys) and cysteinylglycine (CysGly), in plasma interacts via redox and disulfide reactions becoming part of a dynamic system referred to as redox thiols status, which is linked to the antioxidant defense system [20].

Aim of this study was to evaluate the efficacy of the three different treatments on lowering Hcy level in subjects with “moderate” hyperhomocysteinemia particularly comparing traditional 5MTHF pharmacological supplementation, to others supplementations where a low quantities of folic acid were combined or not with Oxo. Secondary aim was to evaluate the effect of the different treatments on redox status of thiols.

## 2. Materials and Methods

One hundred sixty-nine individuals were recruited by an intranet call inside Niguarda Ca’ Granda Hospital (Milan, Italy). Participants were screened on the basis of their Hcy level. Hyperhomocysteinemia (HHcy) is defined when plasma level exceeds 15 μmol/L [21]. The exclusion criteria for this study were Hcy levels <15 and >25 μmol/L, smoking, pregnancy, cancer and regular use of drugs that inhibit the Hcy metabolism (methotrexate, anti-epileptic drugs and others). Thirty-two participants, deemed to be enrolled, gave their written informed consent before being included. This study was conducted in accordance with the Good Clinical Practice guidelines and the Declaration of Helsinki. The protocol was approved (13 March 2009, 63/03_2009) by Niguarda Ca’ Granda Hospital Ethics Committee.

### 2.1. Study Design

Subject anthropometric measures (height and weight) and body mass index (BMI), based on the (WT·(HT)^−2^; kg·m^−2^) formula, were recorded. The thirty-two moderate-HHcy patients (age 48 ± 14; 16 male and 16 female) were distributed into three double-blind randomized homogeneous groups: one group was supplemented with 5 mL/day of oxoproline alone (magnesio+); another group was supplemented with 5 mL/day of oxoproline combined with 300 μg folic acid (oxifolic); the last one was treated with standard therapy (traditional: prefolic (5-methyl-tetrahydrofolate: 5-MTHF) 15 mg/day). magnesio+ and oxifolic used in the study (Table 1) were supplied by DRIATEC (Italy). All participants were advised not to use any other folate or vitamin supplements during the study.

The observation period lasted three months. At enrolment (0 day), after 30 days, and again after 90 days on each patient a blood sample collection was performed to monitor the Hcy levels. Venous blood samples were drawn from an antecubital vein and collected in heparinized and EDTA tubes (Becton Dickinson Company, Oxford, UK). Blood samples were obtained between 08:00 A.M. and 10:00 A.M. after an overnight fast. Concomitant treatments prescribed by their primary physician were continued during the study and recorded at each visit. Study participants were asked to notify the investigators of any adverse events which occurred during the trial and were also asked to avoid every change in lifestyle and dietary regimens.

### 2.2. Homocysteine and Aminothiols Assay

All blood samples were centrifuged at 4000 rpm within 30 min from blood collection; plasma was stored in multiple aliquots and frozen at −20 °C until analysis. Hcy and thiols analysis was performed by a HPLC method previously published [22]. Plasma total aminothiols were measured according to methods validated in our laboratory [23]. Plasma (100 μL) was mixed with 10 μL of tri-n-butylphosphine 10% (*v/v*) in dimethylformamide. After incubation of 30 min at 4 °C, 100 μL of 10% trichloroacetic acid (1:1 *v/v*) were added, shaken and centrifugated for 2 min at 10.000 rpm. To 100 μL of surnatant, 100 μL of 1-mol/L borate buffer pH 11 containing 4-mmol/L EDTA, 10 μL of 1.55-mol/L NaOH and 10 μL of ammonium-7-fluorobenzo- 2-oxa 1,3-diazole-4-sulfonate (10-mg/mL in borate buffer 1-M pH 9.5) were added. The mixture was incubated 60 min at 60 °C in the dark before chromatographic analysis. The fluorescent products were injected (10 μL) in a HPLC isocratic system.

Thiols separation was performed by isocratic high-performance liquid chromatography analysis on a Discovery C-18 column (250 × 4.6 mm I.D, Supelco, Sigma-Aldrich), eluted with a solution of 0.1-mol/L potassium dihydrogenphosphate–acetonitrile (92:8, *v/v*), pH 2.1 at a flow rate of 1 mL/min, as previously described [18]. Fluorescence intensities were measured with an excitation at 385 nm and emission at 515 nm, using a Jasco fluorescence spectrophotometer. Eluted thiols’ peaks had characteristic retention times: Cys—4.2 min; CysGly—5.0 min; Hcy—6.5 min; GSH—8.0 min. No internal standards were utilized for this analysis, as previous described [23].

The chromatographic peak of aminothiols was integrated by dedicated software, and the concentrations were determined with a calibration curve.

### 2.3. Sample Size and Statistical Analysis

Data are expressed as mean ± SD and were analyzed using parametric statistics following mathematical confirmation of a normal distribution using Shapiro-Wilks *W* test. Experimental data were compared by ANOVA variance analysis followed by Tukey’s multiple comparison test to further check the among group significance. A *p*-value of <0.05 was considered statistically significant. Change Δ% estimation [((post value-pre value)/pre value) * 100] is also reported in the text. Statistical analyses were performed using the software Prism 8 (GraphPad Prism 8.3, Software, Inc., San Diego, CA, USA). The prospective calculation of the sample size was determined choosing the value of homocysteine (GPower 3.1) [24].

## 3. Results

The demographic characteristics of the study population and the variables measured at baseline are shown in Table 2. At the time of the enrolment, all 3 groups did not significantly differ for age, weight, gender, BMI and Hcy values (Table 2).

**Figure 2:**
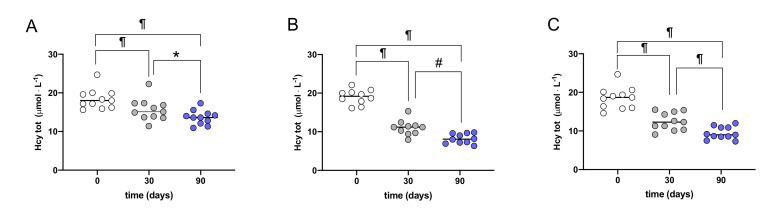
Plots of Hcy levels, according to group after 30 (gray symbols) and 90 (blue symbols) days of supplementation of (**A**) magnesio+; (**B**) oxifolic; (**C**) traditional in patients with baseline moderate HHcy. Data are displayed as mean ± SD. *p*-values refer to 30 and 90 days compared to the baseline and 30 vs. 90 days and are represented by symbols as follows: * *p* < 0.05; # *p* < 0.01; ¶ *p* < 0.0001.

Reduction of Hcy levels, according to group after 30 and 90 days of supplementation in patients with baseline moderate HHcy, are presented in Figure 2.

Every supplementation induced significant (*p* range <0.05–0.0001) reductions of Hcy concentration both at 30 days and larger at 90 days. Hcy level post 90 days magnesio+ supplementation resulted significantly higher than after oxifolic (*p* > 0.0001) and traditional (*p* < 0.005) supplementation. No significant difference in Hcy level post 90 days between traditional and oxifolic supplementation was observed.

In Table 3, the values of total aminothiols concentration recorded at baseline (pre) and after 90 days (post) of the different type of supplementation are summarized. No significant difference in aminothiols concentration at pre and post between the three groups of subjects was observed.

Significant decreases were detected in Cys concentration after traditional (*p* < 0.01), oxifolic and magnesio+ (*p* < 0.05) supplementation. Otherwise GSH concentration significantly increased after oxifolic (*p* < 0.01) and traditional (*p* < 0.05) supplementation.

## 4. Discussion

This study reports results of a clinical trial which investigates the efficacy of simultaneous supplementation with oxoproline and low folic acid doses in decreasing moderate high Hcy levels.

Because HHcy may be associated with pathologic conditions, the reduction of Hcy levels becomes important. Indeed, chronic hyperhomocysteinemia causes vascular remodeling by instigating vein phenotype in artery thus leading to cerebrovascular and vascular dysfunctions [25,26]. The risk of mortality increases for each 5-μmol/L Hcy by 33.6% [27]. The development of neurodegenerative diseases including Alzheimer’s disease can be directly attributed to neurotoxic effect of Hcy [28,29,30,31]. Moreover, a marked increase in the incidence and prevalence of age-associated diseases in concomitance with elevated homocysteine levels [32] may develop bone fractures, poor wound healing, loss of regenerative ability, cardiovascular dysfunction and decline in renal and cognitive functions [33].

Folic acid supplementation is known to be effective in the reduction of Hcy levels. In humans, increased folic acid intake leads to elevated blood concentrations of naturally occurring folates and of unmetabolized folic acid that unfortunately may be related to decreased natural killer cell cytotoxicity. Moreover, in the elderly, a combination of high folate levels and low vitamin B-12 status may be associated with an increased risk of cognitive impairment and anemia and, in pregnant women, with an increased risk of insulin resistance and obesity in their children. On cancer folate has a dual effect protecting against cancer initiation but facilitating progression and growth of preneoplastic cells and subclinical cancers.

Modest increases in cellular concentrations of folates will activate several folate-dependent enzymes, whereas large increases in concentrations may inhibit these and related enzymes [34].

Therefore, it results important that a dose as low as 300 μg of folic acid, in association with oxoproline (oxifolic), was effective in reducing Hcy levels (−57%) such as the conventional dose of 15 mg 5-MTHF (−49%). Our data are in agreement with other studies showing that a folic acid supplementation up to 200 µg/day by 5-MTHF, or folic acid may result as effective in reducing Hcy concentration as supplementation at higher levels [35,36,37]. Anyway, to the best of our knowledge oxifolic supplementation has resulted more effective in lowering plasma total Hcy than every others supplementation previously adopted [38]. Moreover, more important in our study is the observation that patients treated with magnesio+ showed a significant decrease in Hcy levels, albeit somewhat lower (−26%). The finding that oxoproline alone was weakly, but significantly, effective in reducing Hcy levels was in line with the hypothesis of the study. Indeed, this is probably owing to the function of Oxo in facilitating the synthesis of glutamic acid and resulting in an increase of the polyglutamate–folates which is an indispensable condition for the intracellular trapping of folic acid. These findings may indicate thereafter an increased availability of the endogenous pool of folic acid by the supplementation with oxoproline.

Impaired homocysteine metabolism is associated with oxidative stress, inactivation of nitric oxide synthase pathway (including expression, localization, activation and activity) and mitochondria dysfunction leading to tissue degeneration. One possible mechanism of induction of oxidative stress is linked to Hcy thiol group which rapidly undergoes autoxidation in presence of oxygen and metal ions. Moreover, hyperhomocysteinemia promotes Nicotinamide Adenine Dinucleotide Phosphate (NADPH) oxidase activity with increase in Reactive Oxygen Species generation. Oxoproline is an intermediate of the γ-glutamyl cycle that synthesizes reduced glutathione. This production associated with those coming from transsulfuration pathway of Hcy may result in an enhancement of redox status.

Cysteine, the more abundant amino thiol in human plasma and a substrate for the synthesis of GSH, although less reactive than Hcy, promotes detachment of human arterial endothelial cells in culture [39] Cys exhibits auto oxidation properties in the presence of metal ions, thus producing free radicals and in vitro hydrogen peroxide [40]. Thereafter also the significant reduction of Cys level, observed after all type of supplementation adopted in the study, may result in a beneficial effect.

Moreover, the decreased Cys levels found could be related to the significant increased GSH synthesis and turnover generated by a more efficient intracellular flux of substrates through the methionine-Hcy cycle. Considering our data, we may hypothesize a more efficient trans sulfuration pathway.

Some limitations of our study need to be taken into account. As a result of limited funding, we did not measure the plasma and erythrocytes unmetabolized folic acid levels; therefore, further studies are required. Moreover the study provides no information on the effect of treatments with oxoproline on neither glutamate nor polyglutamate–folates. Indeed, an appropriate assessment of these metabolites needs of cellular samples which collection had not been authorized by Ethics Committee.

## 5. Conclusions

The integration of oxoproline which harmless is based on natural presence in food, production by our body and kinetics of metabolic pathway, at dose lower that three grams per day as certified by European Food Safety Authority, resulted an interesting alternative to the traditional therapy for HHcy. This probably because of the consequent intracellular synthesis of glutamic acid and polyglutamate. Absence of folates in the integrator eliminates any chance of excess in unmetabolized folic acid that can constitute a long-term risk. However, further studies are needed to shed light on the findings of the present study. However, it remains unclear whether correction of hyperhomocysteinemia is able to prevent the development of vascular disease, homocysteine-lowering B vitamins and antioxidant therapy may be useful in lowering the risk of inflammation and vascular risk factors.

## Figures and Tables

**Table 1:** Daily dose composition of commercial integrator by Driatec.

	Magnesio+	Oxifolic
Magnesium pidolate	588 mg	652 mg
Magnesium	57 mg	
Oxoproline	538 mg	595 mg
Folic acid	–	300 μg

**Table 2:** Anthropometric and demographic characteristics from all subjects of the different experimental groups. Data are presented as mean ± SD. All formulations tested were well-tolerated. No major or minor adverse reactions were reported.

Therapy	Sex (Male)	Age (Years)	Hight (cm)	Weight (kg)	BMI (kg·m^−2^)	Hcy (μM)
Magnesio+	5 (46%)	50 ± 19	168 ± 8	73.4 ± 9.7	26.2 ± 3.6	18.4 ± 2.6
Oxifolic	5 (50%)	48 ± 9	176 ± 5	81.4 ± 9.6	26.5 ± 4.1	19.0 ± 1.9
Traditional	6 (55%)	46 ± 13	171 ± 10	73.1 ± 12.7	24.9 ± 4.1	18.5 ± 2.8

**Table 3:** Mean (±SD) concentration in μmol·L^−1^ of plasma total aminothiols at baseline (pre) and after 90 days (post) supplementation. Significant differences compared to pre *p* < 0.05 (* symbol), *p* < 0.01 (# symbol), ¶ *p* < 0.0001.

Thiols	Magnesio+		Oxifolic		Traditional	
**μmol·L^−1^**	**Pre**	**Post**	**Pre**	**Post**	**Pre**	**Post**
**Cys**	267.8± 48.0	216.8 ± 40.8 *	259.7± 61.9	203.1 ± 37.8 *	266.2 ± 54.3	215.9 ± 55.7 #
**Hcy**	18.4 ± 2.6	13.6 ± 1.8 ¶	19.0 ± 1.9	8.2 ± 1.2 ¶	18.5 ± 2.8	9.5 ± 1.6 ¶
**CysGly**	26.9 ± 5.0	27.9 ± 5.0	25.3 ± 4.5	25.8 ± 4.7	28.1 ± 4.5	29.4 ± 4.1
**GSH**	4.99 ± 1.77	6.08 ± 1.29	5.04 ± 0.88	6.85 ± 2.13 #	4.71 ± 0.61	6.30 ± 2.18 *
